# Draft Genome Sequences of Two Historical *Listeria monocytogenes* Strains from Human Listeriosis Cases in 1933

**DOI:** 10.1128/genomeA.01364-16

**Published:** 2016-12-08

**Authors:** Vikrant Dutta, Sangmi Lee, Todd J. Ward, Nathane Orwig, Eric Altermann, Dereje Jima, Cameron Parsons, Sophia Kathariou

**Affiliations:** a Department of Food, Bioprocessing and Nutrition Sciences, North Carolina State University, Raleigh, North Carolina, USA; b Center for Human Health and the Environment, North Carolina State University, Raleigh, North Carolina, USA; c Bioinformatics Research Center, North Carolina State University, Raleigh, North Carolina, USA; d USDA-ARS, Peoria, Illinois, USA; e Rumen Microbiology, AgResearch Grasslands, Palmerston North, New Zealand; f Riddet Institute, Massey University, Palmerston North, New Zealand

## Abstract

We report here the draft genome sequences of two *Listeria monocytogenes* strains from some of the earliest reported cases of human listeriosis in North America. The strains were isolated in 1933 from patients in Massachusetts and Connecticut, USA, and belong to the widely disseminated hypervirulent clonal complex 1 (CC1) and CC2.

## GENOME ANNOUNCEMENT

*Listeria monocytogenes* is a facultative intracellular foodborne pathogen widely distributed in the environment and responsible for listeriosis, which can have severe symptoms, including septicemia, stillbirths, and meningitis, and carries an approximately 20% case fatality rate (1, 2). Most human listeriosis cases result from the consumption of foods contaminated in food-processing facilities and involve strains of serotype 1/2a, 1/2b, or 4b (3, 4). Since 2013, whole-genome sequencing (WGS) has been used routinely to analyze *L. monocytogenes* from foods and clinical cases in the United States. However, there is a dearth of WGS data for earlier isolates implicated in human listeriosis, especially “historical” isolates from the pre-World War II era. We report here the draft genome sequences of two such historical isolates of *L. monocytogenes* serotype 4b. *L. monocytogenes* strains OLM10 (child, MA) and OLM11 (infant, CT) were both isolated in 1933, representing some of the earliest reported cases of human listeriosis in North America. Both strains are members of hypervirulent, widely disseminated clones. *In silico* analysis indicates that OLM10 has sequence type 1 (ST1), belonging to the hypervirulent clonal complex 1 (CC1) (5), as did another historical listeriosis isolate recently sequenced (6). OLM11 has ST48 and is a member of another hypervirulent clone, CC2 (5), representing, to our knowledge, the earliest human strain of this clone to yield WGS data.

Genomic DNA was extracted using DNeasy blood and tissue kit (Qiagen, Valencia, CA). Sequencing libraries were prepared using the NEBNext Fast DNA library prep set for Ion Torrent (New England BioLabs, Ipswich, MA) on a Zephyr NGS workstation (PerkinElmer, Waltham, MA). Sequencing was performed on an Ion Torrent Personal Genome Machine using an Ion 318 Chip version 2 and the Ion PGM 400 sequencing kit (Life Technologies, Grand Island, NY). Sequencing produced 6,175,875 reads, with a median read length of 282 nucleotides (nt). Reads were deconvoluted based on barcode sequences attached during library preparation and quality trimmed and *de novo* assembled using the CLC Genomics Workbench 7.5.1 software (CLC bio, Boston, MA), with default parameters. The assembly size was ca. 2.8Mbp, with average coverages of ca. 43× and 63× with 72 and 153 contigs for OLM10 and OLM11, respectively. Annotations were performed using an updated version of the GAMOLA annotation suite (7). Genome annotations identified 3,020 and 3,143 coding sequences, 14 and 12 rRNAs, and 59 and 58 tRNAs for OLM10 and OLM11, respectively.

The genome sequences in this announcement can be used for comparative genomic analyses of *L. monocytogenes* from different periods and regions, and for assessments of genomic stability of hypervirulent clones from human listeriosis.

### Accession number(s).

This whole-genome shotgun project has been deposited in DDBJ/ENA/GenBank under the accession numbers MIMA00000000 and MIMB00000000. The versions described in this paper are the first versions.
